# Supplementary material for: Type 2 Cystatins and Their Roles in the Regulation of Human Immune Response and Cancer Progression
Source: Cancers (Basel). 2023 Nov 10;15(22):5363. doi: 10.3390/cancers15225363 (PMC10670837; doi:10.3390/cancers15225363)
Supplement: Supplementary file 1 [file cancers-15-05363-s001.zip › cancers-2651878-supplementary.pdf]

Supplementary Materials:

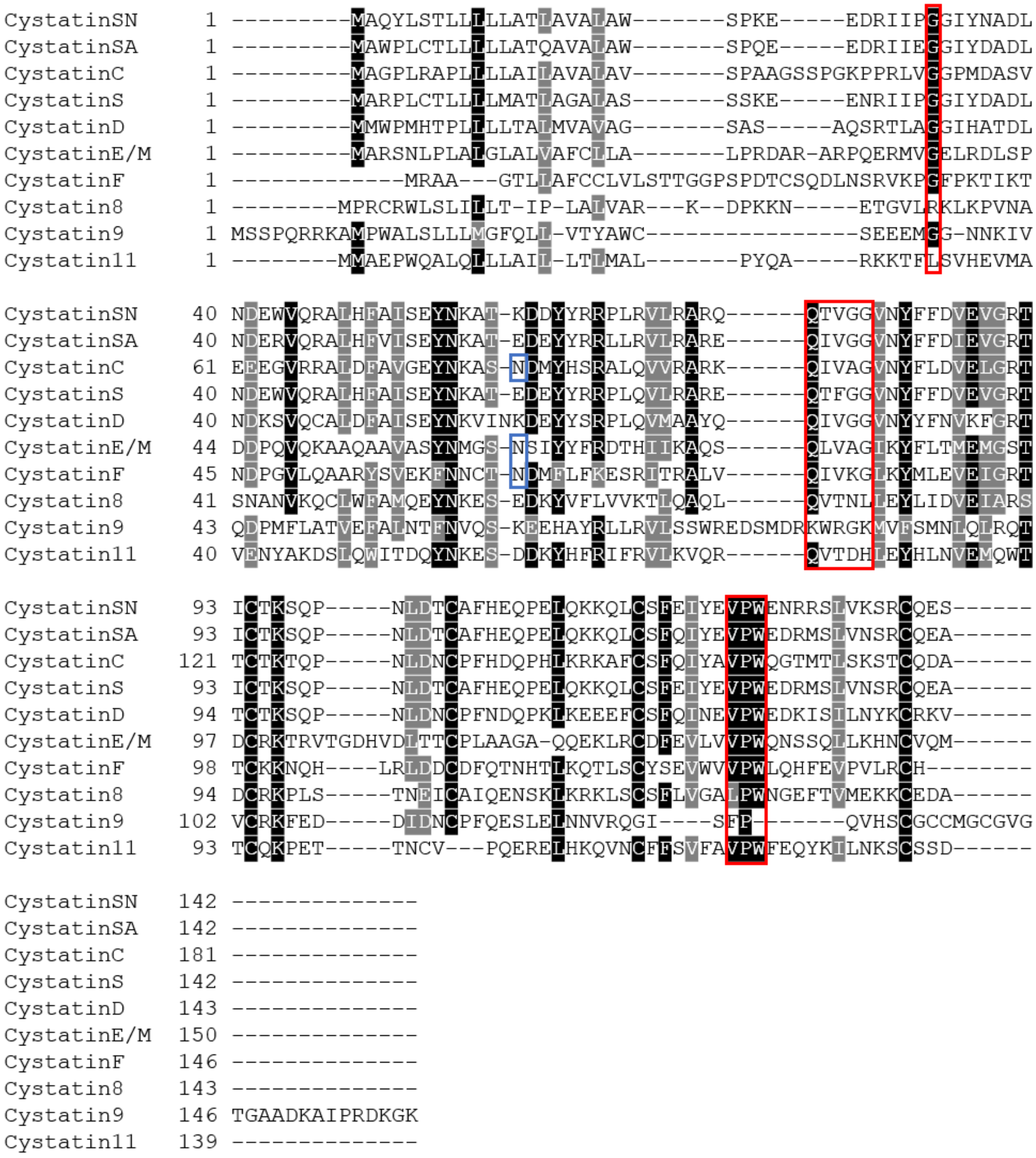

Figure S1: The sequence alignment of type 2 cystatin family. The sequence of each cystatin was retrieved from the NCBI GenPept database. Multiple sequence alignment is done by Clustal Omega and Boxshade. The G-QXVXG-VPW fragment is highlighted in the red box and post-helix N is highlighted in the blue box.

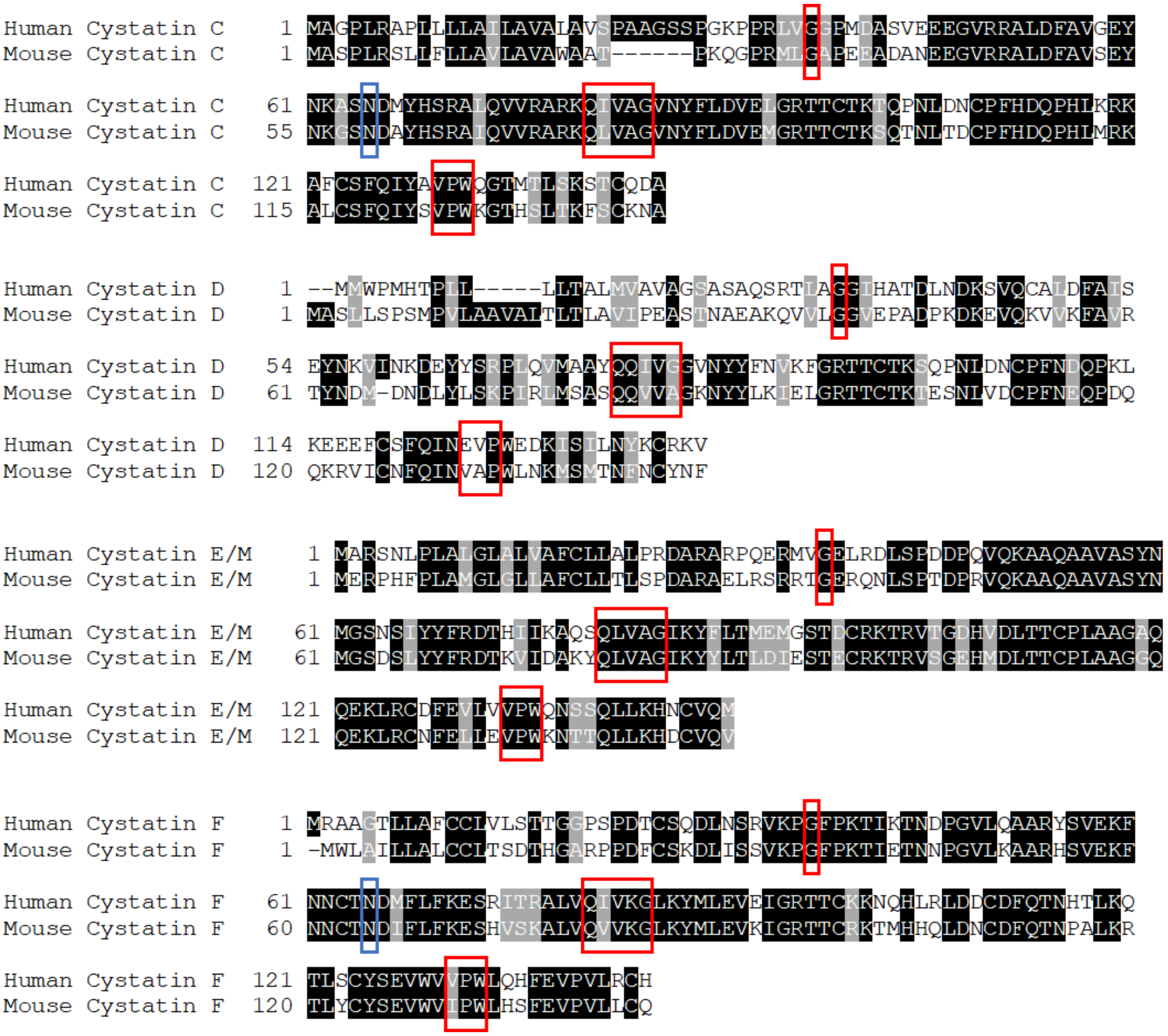

Figure S2: The sequence alignment of human and mouse cystatin C, D, E/M, and F. The sequence of each cystatin was retrieved from the NCBI GenPept database. The sequence alignment is done by Clustal Omega and Boxshade. The G-QXVXG-VPW fragment is highlighted in the red box and post-helix N is highlighted in the blue box.
